# Systematic Comparison of Structural Characterization of Polysaccharides from *Ziziphus Jujuba cv. Muzao*

**DOI:** 10.3390/molecules28020562

**Published:** 2023-01-05

**Authors:** Xiaolong Ji, Shuli Zhang, Xueyuan Jin, Chuanxue Yin, Yang Zhang, Xudan Guo, Ximeng Lin

**Affiliations:** 1 Henan Key Laboratory of Cold Chain Food Quality and Safety Control, Henan Collaborative Innovation Center for Food Production and Safety, College of Food and Bioengineering, Zhengzhou University of Light Industry, Zhengzhou 450001, China; 2 School of Clinical Medicine, Hainan Vocational University of Science and Technology, Haikou 571126, China; 3 Hebei Higher Education Institute Applied Technology Research Center on TCM Development and Industrialization, Hebei TCM Formula Preparation Technology Innovation Center, Basic Medical College, Hebei University of Chinese Medicine, Shijiazhuang 050200, China; 4 College of Food Science and Engineering, Northwest A&F University, Yangling, Xianyang 712100, China

**Keywords:** jujube, polysaccharide, structural characterization, NMR

## Abstract

To investigate the structural information differences of *Ziziphus Jujuba cv. Muzao* polysaccharides, ten samples were successfully extracted from aqueous and alkaline solutions, prepared via DEAE-Sepharose Fast Flow through different eluents and Sephacryl S-300 columns, and systematically analyzed. Their characteristics were studied and then compared using chemical testing, high-performance gel permeation chromatography (HPGPC), gas chromatography (GC), methylation analysis, and NMR spectroscopy. The data achieved demonstrated that different jujube polysaccharide fractions possessed different structural characteristics, and most of them belonged to pectic polysaccharides. Overall, the structural information difference of jujube polysaccharides was preliminarily illuminated, which could not only promote the potential application of *Z. Jujuba cv. Muzao* polysaccharides but also provide an effective way to analyze the structures of polysaccharides from other genera jujube fruit.

## 1. Introduction

Chinese jujube (*Zizyphus Jujuba Miller*), named “Dazao” or “Hongzao” in Chinese, is mainly found in Asia’s subtropical and tropical regions, particularly in the eastern regions, the Yellow River Valley regions, and the northwest [1]. Traditional Chinese medicine (TCM) often employs jujube as a crude medicament since it is a fruit that is advantageous and lucrative. According to Bencao Gangmu, written between 1552 AD and 1578 AD, jujube fruit was believed to improve people’s “Qi” (the vital energy) and “Blood” (body circulation) [2]. Nutritional findings have reported that various functional compositions within jujube are being proposed, including total phenolics, individual phenolic compounds, triterpenic acids, polysaccharides, α-tocopherol, carotene, vitamin C (Vc), nucleosides, nucleobases, etc. [3]. According to pharmacological investigations, jujube has various medical qualities, such as anti-obesity, anticancer, anti-inflammatory and antioxidant, as well as immunostimulating, gastrointestinal protective, and hepatoprotective properties. It also inhibits the production of foam cells in macrophages [3,4,5].

Chinese jujubes, *Ziziphus Jujuba cv. Muzao* (*Z. Jujuba cv. Muzao*), are now often planted in the regions of the Yellow River Valley (Yulin and Lüliang, Shaanxi, China) [6]. Significant interest has emerged in fresh *Z. Jujuba cv. Muzao* fruit because of its high content of water (68%), titratable acid (0.32%), soluble solids (27.4%), total sugar (21.9 g/100 g), Vc reducibility (376.2 mg/100 g), total procyanidins (184.2 mg GSPE eq./100 g FW), total phenol (541.8 mg GAE eq./100 g FW), cinnamic acid (35.2 µg/100 g FW), total flavonoids (255.5 mg Rutin eq./100 g FW), etc., which make them a crucial source of food for people [7,8]. The molecular weight, monosaccharide content, and chemical structures of polysaccharides all affect their bioactivity. According to some reports, *Z. Jujuba cv. Muzao*’s primary bioactive ingredients are polysaccharides for their positive effects on health, including an anti-inflammatory and hypolipidemic effect and antioxidant properties, as well as their ability to improve intestinal barrier function and control intestinal flora [9,10]. *Z. Jujuba cv. Muzao*’s primitive polysaccharides were extracted via ultrasonically aided acidic buffer extraction [11], ultrasonically assisted two-phase aqueous extraction [12], and alkali extraction (0.09 M [13] and 0.1 M). The structural characterization of several polysaccharide fractions extracted from *Z. Jujuba cv. Muzao* has not yet been the subject of a thorough analysis.

In the present study, we emphasized the purification of *Z. Jujuba cv. Muzao* polysaccharides extracted using water (PZMP1, PZMP2-1, PZMP2-2, PZMP3-1, PZMP3-2, and PZMP4) and alkaline (SAZMP1, SAZMP2, SAZMP3, and SAZMP4). Their structural characteristics were examined via high-performance gel permeation chromatography (HPGPC), methylation analysis, gas chromatography (GC), and nuclear magnetic resonance spectroscopy (NMR), which could promote the potential application of jujube polysaccharides to commercial nutritious foods.

## 2. Results and Discussion

### 2.1. PZMPs’ and SAZMPs’ Separation and Purification

**Figure 1:**
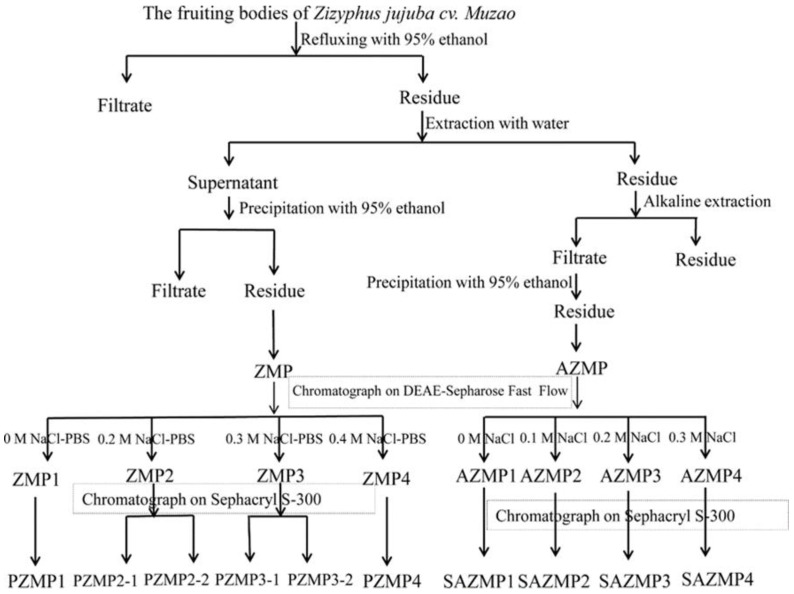
The separation flow diagram of *Ziziphus Jujuba cv. Muzao* polysaccharides.

Deproteinization, extraction with hot water/alkaline extraction, precipitation of ethanol, and freeze-drying were used to produce ZMP and AZMP from *Z. Jujuba cv. Muzao*. Regarding dried jujube fruit power, the overall yield of ZMP and AZMP was 3.82% and 3.30%, respectively. The ZMP and AZMP fractions were progressively separated using Fast Flow (DEAE-Sepharose) and gel filtration columns (Sephacryl S-300) to purify them further. Figure 1 displays a flow diagram of the separation process for ZMP and AZMP fractions. According to the elution curve, PZMP1 and SAZMP1 were determined as neutral polysaccharides, and PZMP2-4 and SAZMP2-4 were acid polysaccharides [14]. The yield was a crucial parameter for extraction efficiency for futural structure research. In a previous study, PZMP yield ranged from 0.76% to 14.61%, with a total yield of 37.64%, based on ZMP level, while SAZMP yield ranged from 2.26% to 10.22%, with a total yield of 24.23%, based on AZMP level [14]. All the total extraction yield of purified ZMP and ZAMP fractions was calculated at 2.24% according to dried jujube fruit power. It is possible that the different eluents and extraction techniques used in this research contributed to the heterogeneity in the yield of jujube polysaccharides [15].

### 2.2. Physical and Chemical Characteristics of PZMPs and SAZMPs

The total carbohydrate, protein content, and phenol of PZMPs and SAZMPs are given in Table 1. Phenol–sulfuric acid assay results revealed that PZMPs and SAZMPs contained higher carbohydrates (>95%), excluding only 92.64% total sugar of PZMP4. The higher the carbohydrate, the more accurately the jujube polysaccharides’ structure could be analyzed [16]. Additionally, traces of total phenol and protein were discovered in all PZMP and SAZMP samples, and only PZMP4 included a higher protein content (3.09%). The result was similar for ZSP4b extracted through *Z. Jujuba cv. Jinsixiaozao*, which might be caused by a higher concentration of NaCl-PBS eluent [15]. The total phenolic contents of SAZMPs were not discovered using the Folin–Ciocalteu reagent technique, and the contents evaluated in PZMPs increased gradually from 0.139% to 0.950%. The ten polysaccharide fractions of *Z. Jujuba cv. Muzao* may have distinct chemical compositions according to these facts.

**Table 1:** Physicochemical property of *Zizyphus jujuba cv. Muzao* polysaccharides.

Samples	Relative Yield (%)	Total Carbohydrate (%)	Protein (%)	Total Phenol (%)	Average Molecular Weight (Da)	Monosaccharide Composition	Structures
PZMP1	2.95	96.12	1.15	0.139	1.697 × 10^4^	Ara, Gal, Glc, Man, Xyl in the ratio of 17.36:3.29:2.68:1.05:1.00	Backbone composed of (1→3,5)-linked-Ara*f*, (1→3)-linked-Ara*f*, (1→5)-linked-Ara*f*, (1→4)-linked-Glc*p*, and (1→)-linked-Ara*f*, (1→)-linked-Glc*p*. With branches attached to *O*-3 and *O*-5 of some residues.
PZMP2-1	2.13	95.58	2.04	0.210	1.410 × 10^5^	Rha, Ara, Gal, Gal*p*A in the ratio of 0.84:5.88:0.31:0.12	Backbone composed of→5)-a-L-Ara*f*-(1→, and→3,5)-a-L-Ara*f*-(1→residues, with branches attached to *O*-3 atom of arabinose, and terminated with Ara*f*.
PZMP2-2	14.15	96.92	0.69	0.217	6.273 × 10^4^	Rha, Ara, Gal, Xyl, Gal*p*A in the ratio of 1.18: 5.23: 2.68: 0.22: 2.20	Backbone composed of (1→4)-linked Gal*p*A and (1→2,4)-linked Rha*p* residues, with branches at the *O*-4 position, consisting of Ara*f* and Gal*p* residues.
PZMP3-1	3.04	95.35	2.11	0.320	2.410 × 10^5^	Rha, Ara, Gal, Gal*p*A in the ratio of 2.56:7.70:3.73:6.73	Backbone composed of (1→2,4)-linked Gal*p*A, (1→4)-linked Gal*p*A, (1→4)-linked Gal*p*, (1→3)-linked-Ara*f*, (1→5)-linked-Ara*f* and (1→)-linked-Ara*f*.
PZMP3-2	14.61	96.56	0.69	0.145	5.821 × 10^4^	Rha, Ara, Gal, Gal*p*A in the ratio of 1.74:2.00:1.00:18.69	Backbone composed of→4)-GalpA-(1→, with few branches at the *O*-2 position of some Ara*f* and Rha*p* residues.
PZMP4	0.76	92.64	3.09	0.950	2.790 × 10^4^	Rha, Ara, Gal, Glc, Man, Gal*p*A in the ratio of 2.32:2.21:2.08:0.88:0.22:8.83	Backbone composed of (1→4)-linked Gal*p*A with three branches bonded to *O*-3 of (1→3)-linked Ara*f*, (1→2)-linked Rha*p*, and terminated with Gal*p*A.
SAZMP1	2.26	-	2.01	nd	8.910 × 10^3^	Rha, Ara, Gal, Glc, Man in the ratio of 0.97:13.82:11.85:71.21:2.15	-
SAZMP2	2.97	-	1.91	nd	8.230 × 10^3^	Rha, Ara, Gal, Glc, Man, Xyl, Gal*p*A in the ratio of 2.13:65.06:17.96:1.39:0.39:3.35:9.72	-
SAZMP3	8.78	96.80	0.96	nd	9.730 × 10^3^	Rha, Ara, Gal, Glc, Man, Xyl, Gal*p*A in the ratio of 10.51:6.70:0.59:0.26:0.50:74.69	Backbone composed of 1,4-α-D-GalA*p* with side chains of 1,3-*β*-D-Gal*p*, 1,3,5-linked Ara*f*, 1,2,4-α-L-Rha*p* and terminals of 1-linked Ara*f*, 1-linked Rha*p*, 1-linked Gal*p*.
SAZMP4	10.22	96.52	1.11	nd	2.894 × 10^4^	Rha, Ara, Man, Xyl, Gal*p*A in the ratio of 3.24:2.89:0.22:0.17:93.48	Backbone composed of 1,4-linked Gal*p*A with side chains of 1,2,4-linked Rha*p* and 1,3,5-linked Ara*f* and terminals of 1-linked Rha*p* and 1-linked Ara*f*.

nd: not detected. -: no experimental data. PZMP1] [17], PZMP2-1 [18], PZMP2-2 [19], PZMP3-1 [20], PZMP3-2 [21], PZMP4 [22], SAZMP1 [23], SAZMP2 [23], SAZMP3 [24], SAZMP4 [25].

### 2.3. Weight of Molecular and Composition of Monosaccharide Analysis

Jujube polysaccharide molecular weight distribution is essential for biological activity. As shown in Table 1, a wide range of *Mw* (8.23–141 kDa) was found in PZMPs and SAZMPs. Compared with water-extracted jujube polysaccharides, the *Mw* of alkaline (0.1 M NaOH solution) extracted polysaccharides was generally lower, which may be explained by the alkaline solution’s influence on the glycosidic bond’s breakdown, accelerating the destruction of the jujube saccharide molecule [26]. Low *Mw* jujube polysaccharides may have significant potential benefits for intestinal absorption, as well as for food digestion and the development of intestinally beneficial bacteria [16]. Different molecular weights of ZMP were attributed to different extraction conditions [10].

Information on the monosaccharide composition is far more crucial for understanding jujube polysaccharides’ physiochemical and structural characteristics. As can be seen in Table 1, almost all of the PZMPs and SAZMPs were made up of seven distinct types of monosaccharides with varying amounts. Rhamnose, galacturonic acid, galactose, and arabinose were among the principal constituents in ZMP and SAZMP samples. Additionally, the structural types of jujube polysaccharides were determined by calculating the relative content ratio of (Ara + Gal)/Rha (0.69–38.98). While PZMP1 and AZMP1 contained no uronic acid, the amount of galacturonic acid assessed in other jujube polysaccharide fractions rose steadily, which was connected with the rising ion exchanging capacity of the NaCl eluent [14]. These data reconfirmed that different purified jujube polysaccharides possessed other structural characteristics, and SAZMP4 contained the most acidic group.

### 2.4. Analysis of Methylation

Determining glycosyl linkages in jujube polysaccharides may be achieved using GC-MS to examine methylation, which is a reliable and effective method. The findings are shown in Table S1 based on the CCRC Spectral Database’s default data. The following conclusions can be drawn: (1) Different PZMPs and SAZMPs had several primary forms of sugar bonds, and the most prevalent derivatives matched the monosaccharide composition discovered by GC. (2) 1,3,5-, 1,5- and 1,3-linked Ara*f* were the central glycosidic bonds in all ZMP and AZMP samples. (3) In the PZMPs, the galacturonic acid proportion increased with the concentration of eluent sodium chloride [14]. No uronic acid was found in the AZMPs (SAZMP3 and SAZMP4), which might be because the alkaline solution reacted with the acidic group of uronic acid and could not be detected [27]. (4) The fact that the total amount of terminal Ara*f*/Gal*p*/Glc*p*/Rha*p* residues was about equivalent to the total amount of branching residues indicates that all PZMPs and SAZMPs underwent successful methylation.

### 2.5. Analysis of NMR

NMR analysis was performed on the PZMPs and SAMPs to shed light on their structural details further. Table S2 summarizes all the relevant NMR data for various glycosidic connections. The following conclusions could be made: (1) Pure jujube polysaccharides had various main anomeric proton signals; 3–8 main anomeric proton signals were found in the PZMPs. (2) The protons in the C-residues with chemical shifts between 5.30 and 3.60 ppm in the ^1^H NMR spectra were identified. (3) The same glycosidic bond types might have different C/H proton signals under the same/different NMR conditions. (4) The NMR glycosidic bonds of C/H signal assignment were similar to methylation results. (5) Through NMR and methylation analysis, the structural characteristics and structural types of jujube polysaccharides could be determined, and most of the jujube polysaccharides belonged to pectin polysaccharides [28,29].

## 3. Materials and Methods

### 3.1. Chemicals and Materials

Loess Plateau Experimental Orchard (Yulin, Shaanxi, China) provided the fruit bodies of *Z. Jujuba cv. Muzao*. GE Healthcare Life Sciences (Piscataway, NJ, USA) offered Sephacryl gels (S-300) and anion-exchange Sepharose Fast Flow (DEAE). Sigma Chemical Co. (St. Louis, MO, USA) provided standard monosaccharides. The remaining substances were all of analytical grade.

### 3.2. Isolation and Purification of PZMPs and SAZMPs

Fresh jujube fruit was meticulously cleaned, cut in half, stoned, and dried in an oven before being powdered and put through a sieve with a mesh size of 60. Then, 95% ethanol was used to pre-extract the fat, color, and sugars with low molecular weight [10]. Jujube powder was used to extract the polysaccharides, which were then concentrated, filtered, and mixed with 95% ethanol to create *Z. Jujuba cv. Muzao* polysaccharides (ZMP). An alkaline solution was used to treat the insoluble residues, and the alkaline extracted supernatants were combined with 95% ethanol to create ZMP (AZMP). Then, ZMPs and AZMPs were decolorized and deproteinized. The ZMP/AZMP solutions were put onto a Fast Flow column (DEAE-Sepharose), sequentially eluting the column with a gradient of NaCl solutions (0 and 0.2–0.4 M)/(0–0.3 M) and phosphate-buffered saline (PBS, 20 mM, pH 6.0) and at 1.5/1.2 mL per minute flow rate, to obtain ZMP fractions (PZMP1-4)/AZMP fractions (AZMP1-4). Following that, utilizing a gel-permeation chromatography column (Sephacryl S-300), each fraction was cleaned, which was eluted using deionized water at a 0.8 mL per minute flow rate to collect ZMP fractions (PZMP1 [17], PZMP2-1 [18], PZMP2-2 [19], PZMP3-1 [20], PZMP3-2 [21], and PZMP4 [22])/AZMP fractions (SAZMP1 [23], SAZMP2 [24], SAZMP3 [24], and SAZMP4 [25]). The total ten fractions (Figure 1) were collected, respectively, then compared for further structural characterization.

### 3.3. Analysis of Chemicals

The phenol–sulfuric acid technique was used to determine the total sugar content of PZMPs and SAZMPs, using glucose as the benchmark [30]. Using the Bradford technique and the reference concentration of bovine serum albumin, the amount of protein was measured [31]. With minor modifications, gallic acid was utilized as the reference material to determine the total phenolic content using the Folin–Ciocalteu reagent [32].

### 3.4. Gel-Permeation Chromatography and Monosaccharide Analysis

PZMPs and SAZMPs were identified as monosaccharides and quantified using GC analysis, as previously mentioned [33]. The monosaccharide derivatized product was determined using GC (Shimadzu 2014 C, Kyoto, Japan) and a high-performance capillary column (DB-17, 30 mL × 0.25 mm ID, film thickness of 0.25 μm, Agilent, Santa Clara, CA, USA).

PZMPs and SAZMPs were analyzed using HPLC utilizing Agilent-LC 1200 equipment and a TSK-gel G3000PWxl/G5000PWxl column (7.8 mm × 300 mm) to establish average molecule weights and uniformity [34]. A calibration curve was used to estimate the molecular weights of PZMPs and SAZMPs compared to dextran.

### 3.5. Analysis of Methylation

The reduction methods methylated the samples containing uronic acid. With NaBD_4_ and EDC (1-ethyl-3-(3-dimethyl aminopropyl) carbodiimide), PZMPs/SAZMPs were carboxyl reduced [35]. The stated papers were followed for the methylation analysis procedure [36]. All ZMP and AZMP fractions were further processed via acetylation with acetic anhydride, reduction with NaBH_4_, and hydrolysis with trifluoroacetic acid. Using GCMS-QP2010A equipment with (PZMP1, PZMP3-2, SAZMP3, SAZMP4), a capillary column ((RTX-50, 30.0 m × 0.25 mm × 0.25 μm) and (PZMP2-1, PZMP3-1, PZMP2-2, PZMP4)) and another capillary column (DB-17MS, 60.0 m × 0.25 mm × 0.25 μm), as well as electron ionization spectra, the resulting partly methylated alditol acetate derivatives were discovered.

### 3.6. NMR Analysis

An NMR spectrometer (AVIII-600, Bruker Corporation; Billerica, MA, USA) was used to acquire the NMR spectra in one and two dimensions of PZMPs. SAZMPs swapped for deuterium were diluted in D_2_O (0.5 mL), and NMR spectra (^1^H and ^13^C) were obtained using a Brucker AVANCE III 500 MHz NMR [37].

## 4. Conclusions

The present study prepared a series of *Z. Jujuba cv. Muzao* polysaccharides through various extraction and purification methods and found some interesting interrelations concerning the physicochemical properties of varying *Z. Jujuba cv. Muzao* polysaccharides. The molecular weight ranged from 8.23 to 141 kDa, and the monosaccharide composition was composed of various molar ratios of rhamnose, arabinose, galactose, galacturonic acid, etc. Arabinofuranosyl had methylated sugar derivatives of *Z. Jujuba cv. Muzao* polysaccharides, which belong to pectic polysaccharides. The present work might methodically aid in a more significant comprehension of the many *Z. Jujuba cv. Muzao* polysaccharide structures and offer a thoroughly relevant bibliography for more structure–function correlations and precise structural details for other species of jujube polysaccharides.

## Data Availability

Data are contained within the article.

## Supplementary Materials

The following supporting information can be downloaded at: https://www.mdpi.com/article/10.3390/molecules28020562/s1, Table S1: Methylation analysis data for *Zizyphus jujuba cv. Muzao* polysaccharides; Table S2: Assignments of ^1^H and ^13^C NMR spectra for *Zizyphus jujuba cv. Muzao* polysaccharides.
