# Supplementary material for: Systematic Comparison of Structural Characterization of Polysaccharides from Ziziphus Jujuba cv. Muzao
Source: Molecules. 2023 Jan 5;28(2):562. doi: 10.3390/molecules28020562 (PMC9866945; doi:10.3390/molecules28020562)
Supplement: Supplementary file 1 [file molecules-28-00562-s001.zip › molecules-2074057-supplementary.pdf]

Systematic Comparison of Structural Characterization of Polysaccharides from *Ziziphus Jujuba cv. Muzao*

Xiaolong Ji [1], Shuli Zhang [1], Xueyuan Jin [2], Chuanxue Yin [1], Yang Zhang [1], Xudan Guo [3,*] and Ximeng Lin [4,*]

[1] Henan Key Laboratory of Cold Chain Food Quality and Safety Control, Henan Collaborative Innovation Center for Food Production and Safety, College of Food and Bioengineering, Zhengzhou University of Light Industry, Zhengzhou 450001, China
[2] School of Clinical Medicine, Hainan Vocational University of Science and Technology, Haikou 571126, China
[3] Hebei Higher Education Institute Applied Technology Research Center on TCM Development and Industrialization, Hebei TCM Formula Preparation Technology Innovation Center, Basic Medical College, Hebei University of Chinese Medicine, Shijiazhuang 050200, China
[4] College of Food Science and Engineering, Northwest A&F University, Yangling 712100, China
* Correspondence: guoxudan123@126.com (X.G.); ximenglin423@nwafu.edu.cn (X.L.)

Table S1. Methylation analysis data for *Zizyphus jujuba cv. Muzao* polysaccharides.

Samples	Peak No.	Retention Time (min)	Methylated Sugars	Linkage Patterns	Relative Amount (mol%)	Ref.
PZMP1	1	18.923	2,3,5-Me_3-Ara*f*	Ara*f*-(1→	16.44	[17]
	2	20.676	2,3,4,6-Me_4-Glc*p*	Gal*p*-(1→	2.55	
	3	24.030	2,4,5- Me_3-Ara*f*	→3)-Ara*f*-(1→	3.76	
	4	25.979	2,3-Me_2-Ara*f*	→5)-Ara*f*-(1→	26.51	
	5	29.170	2,3,4,6-Me_4-Glc*p*	Glc*p*-(1→	4.14	
	6	30.438	2-Me-Ara*f*	→3,5)-Ara*f*-(1→	29.81	
	7	33.280	2,3,6-Me_3-Glc*p*	→4)-Gal*p*-(1→	16.79	
PZMP2-1	1	58.767	2,3,5-Me_3-Ara*f*	Ara*f*-(1→	-	[18]
	2	65.832	2,3-Me_2-Ara*f*	→5)-Ara*f*-(1→	-	
	3	70.291	2-Me-Ara*f*	→3,5)-Ara*f*-(1→	-	
PZMP2-2	1	36.257	2,3,4,6-Me_4-Gal*p*	Gal*p*-(1→	11.52	[19]
	2	40.142	2,4,5- Me_3-Ara*f*	→3)-Ara*f*-(1→	24.97	
	3	56.720	3-Me-Rha*p*	→2,4)-Rha*p*-(1→	11.86	
	4	63.049	2,4,6-Me_3-Gal*p*	→3)-Gal*p*-(1→	19.2	
	5	65.033	2,3,6-Me_3-Gal*p*A	→4)-Gal*p*A-(1→	18.16	
	6	70.285	2-Me-Ara*f*	→3,5)-Ara*f*-(1→	14.47	
PZMP3-1	1	40.086	2,3,4-Me_3-Rha*p*	Rha*p*-(1→	7.45	[20]
	2	40.245	2,4,5-Me_3-Ara*f*	→3)-Ara*f*-(1→	5.72	
	3	41.437	2,3-Me_2-Ara*f*	→5)-Ara*f*-(1→	39.75	
	4	63.049	2,3,6-Me_3-Gal*p*	→4)-Gal*p*-(1→	16.5	
	5	65.037	2,4,6-Me_3-Gal*p*A	→4)-Gal*p*A-(1→	22.93	
	6	66.250	3,6-Me_2-Gal*p*A	→2,4)-Gal*p*A-(1→	7.65	
PZMP3-2	1	17.720	2,3,5-Me_3-Ara*f*	Ara*f*-(1→	6.25	[21]
	2	19.835	1,2,3-Me_3-Rha*p*	→4)-Rha*p*	2.51	
	3	30.543	2,3,6-Me_3-Gal*p*	→4)-Gal*p*-(1→	2.75	

	4	33.186	2,3,6-Me3-Gal*p*A<br>1,2,3,6-Me4-Gal*p*A	→4)-Gal*p*A-(1→<br>→4)-Gal*p*A	84.64	
	5	34.153	3,6-Me2-Gal*p*A	→2,4)-Gal*p*A-(1→	3.85	
PZMP4	1	38.941	3,4-Me2-Rha*p*	→2)-Rha*p*-(1→	18.95	[22]
	2	39.572	2,3,4,6-Me4-Gal*p*A	Gal*p*A-(1→	11.54	
	3	40.142	2,4,5-Me3-Ara*f*	→3)-Ara*f*-(1→	17.56	
	4	66.252	2,6-Me2-Gal*p*A	→3,4)-Gal*p*A-(1→	51.95	
SAZMP3	1	20.344	2,3,5-Me3-Ara*f*	Ara*f*-(1→	3.98	[24]
	2	29.583	2,3,4-Me3-Rha*p*	Rha*p*-(1→	3.75	
	3	30.817	2,3,4,6-Me4-Gal*p*	Gal*p*-(1→	1.99	
	4	31.641	2-Me-Ara*f*	→3,5)-Ara*f*-(1→	2.95	
	5	32.333	3-Me-Rha*p*	→2,4)-Rha*p*-(1→	7.04	
	6	34.850	2,3,6-Me3-Gal*p*	→4)-Gal*p*-(1→	74.98	
	7	35.142	2,4,6-Me3-Gal*p*	→3)-Gal*p*-(1→	5.31	
SAZMP4	1	20.355	2,3,4-Me3-Rha*p*	Rha*p*-(1→	0.5	[25]
	2	26.563	2,3,5-Me3-Ara*f*	Ara*f*-(1→	0.47	
	3	31.915	2-Me-Ara*f*	→3,5)-Ara*f*-(1→	0.46	
	4	34.707	3-Me-Rha*p*	→2,4)-Rha*p*-(1→	0.52	
	5	35.092	2,3,6-Me3-Gal*p*	→4)-Gal*p*-(1→	28.8	

Table S2. Assignments of ^{1}H and ^{13}C NMR spectra for *Zizyphus jujuba cv. Muzao* polysaccharides.

Samples	Residues	Linkage		1	2	3	4	5	6	Ref.
PZMP1	A	→3,5)-Ara*f*-(1→	C	109.95	82.25	84.12	86.57	69.07		[17
			H	5.16	4.30	4.15	3.98	3.96		
	B	→5)-Ara*f*-(1→	C	110.31	82.25	79.15	86.72	69.03		
			H	5.10	4.30	4.15	4.05	3.85		
	C	Ara*f*-(1→	C	112.14	82.99	79.30	85.09	63.94		
			H	5.25	3.77	4.15	4.23	3.9		
	D	→5)-Ara*f*-(1→	C	110.12	82.25	79.30	85.09	69.05		
			H	5.13/5.12	4.30	4.15	4.23	3.85		
	E	→4)-Gal*p*-(1→	C	105.90	73.26	72.24	76.63	75.7	69.05	
			H	4.54	3.67	3.94	3.94	3.75	3.92	
	F	→3)-Ara*f*-(1→	C	109.95	82.25	84.12	86.57	63.94		
			H	5.16	4.30	4.15	3.98	3.92		
PZMP2-1	A	*α*-L-Ara*f*-(1→	C	107.14/107.51	80.92/81.32	76.62	83.93	61.17		[18]
			H	4.99/5.05	4.03/4.20	3.85	3.94	3.63		
	B	→5)-*α*-L-Ara*f*-(1→	C	107.14/107.51	80.92/81.32	76.62	83.93	66.32/66.58		
			H	4.99/5.05	4.03/4.20	3.85	3.94	3.71		
	C	→3,5)-*α*-L-Ara*f*-(1→	C	109.25	82.29	83.95	83.93	66.32/66.58		
			H	5.14	4.12	3.99	3.94	3.71		
PZMP2-2	A	→3)-Ara*f*-(1→	C	109.97	79.43	86.72	84.13	63.95		[19]
			H	5.16	3.96	4.04	4.14	3.84		
	B	→3)-Ara*f*-(1→	C	110.29	81.91	85.10	84.13	63.95		
			H	5.10	4.30	4.11	4.14	3.84		
	C	→3,5)-Ara*f*-(1→	C	112.10	84.20	86.76	84.13	69.05		
			H	5.25	4.23	4.05	4.14	3.85		
	D	→3)-Gal*p*-(1→	C	103.28/102.97	70.81	81.61	71.52	74.14	63.95	

			H	4.97/4.91	3.74	4.47	4.02	4.72	3.84	
	E	→4)-Gal*p*A-(1→	C	101.81	70.81	81.20	71.52	74.14	178.31	
			H	5.07	3.74	4.39	4.02	4.72		
	F	→3)-Gal*p*-(1→	C	102.32	70.81	81.20	71.52	74.14	63.95	
			H	5.12	3.74	4.39	4.02	4.72	3.84	
	G	→2,4)-Rha*p*-(1→	C	106.01	72.74	74.22	82.97	65.35	23.07	
			H	4.54	3.67	4.79	3.76	3.57	2.08	
	H	Gal*p*-(1→	C	99.00	70.81	85.10	71.52	74.14	63.95	
			H	3.74	3.74	4.11	4.02	3.50	3.84	
PZMP3-1	A	→4)-Gal*p*A-(1→	C	103.39	68.08	71.26	81.37	73.49	170.91	[20]
			H	5.21	3.77	4.08	4.42	4.80		
	B	→2,4)-Gal*p*A-(1→	C	103.39	70.62	71.98	81.37	73.49	171.01	
			H	5.21	3.97	4.10	4.42	4.80		
	C	→3)-Ara*f*-(1→	C	107.53	82.26	84.04	86.79	63.93		
			H	5.12	4.28	4.18	3.98	3.92		
	D	→5)-Ara*f*-(1→	C	109.35	82.26	79.06	86.79	68.34		
			H	5.12	4.28	4.17	3.99	3.93		
	E	→4)-Gal*p*-(1→	C	100.52	70.56	80.96	72.01	74.39	63.67	
			H	4.94	3.71	4.50	4.07	4.77	3.85	
PZMP3-2	A	→4)-*α*-D-Gal*p*A-(1→	C	102.43/101.96	70.88	71.54	80.84/81.33	74.19	173.62	[21]
			H	5.15/5.09	3.75	4.00	4.46	4.70/4.78		
	B	→4)-*β*-D-Gal*p*A-(1→	C	103.35/103.07	70.88	71.66	81.98/81.67	73.67	173.74	
			H	4.96/4.91	3.75	4.00	4.38	4.43		
	C	→4)-*α*-D-Gal*p*A	C	95.13	70.88	71.54	80.84	74.19	173.87	
			H	5.29	3.82	4.00	4.13	4.27		
	D	→4)-*β*-D-Gal*p*A	C	99.07/98.96	70.88	71.54	81.33	77.08	173.87	
			H	4.56/4.57	3.75	4.00	4.13	4.27		
	E	*α*-Ara*f*-(1→	C	110.37	81.33	77.76	85.10	64.01		
			H	5.25	4.33	4.06	4.29	3.73		
	F	→2,4)-*α*-D-Gal*p*A-(1→	C	102.43/101.96	79.47	73.21	80.84/81.33	71.54	173.62	
			H	5.20/5.15	3.98	4.21	4.43	4.00		
PZMP4	A	→3,4)-*α*-D-Gal*p*A-(1→	C	101.89	70.79/70.93	79.42	84.05	73.41/73.04	173.68/173.80	[22]
			H	4.87	3.74	3.87	4.04	5.06/5.00		
	B	*α*-D-Gal*p*A-(1→	C	102.36	71.45	70.79	71.59	74.21	178.21	
			H	5.00	3.91	3.63/3.66	3.96	4.32		
	C	→2)-*α*-L-Rha*p*-(1→	C	102.36	84.19	71.45	74.85	71.60		
			H	4.82	4.11/4.04	3.86	3.66	3.96		
	D	→3)-*α*-L-Ara*f*-(1→	C	110.24	81.61	86.77	83.74	63.96		
			H	5.00/5.05	4.20	3.94	4.02	3.62		
SAZMP3	A	→2,4)-*α*-L-Rha*p*-(1→	C	97.67	82.57	72.23	82.55	74.84	16.78	[24]
			H	5.21	3.74	4.08	3.68	3.19	1.22	
	B	→4)-*α*-D-Gal*p*A-(1→	C	99.16	69.67	69.06	78.09	71.51	175.54	
			H	5.03	3.67	3.96	4.38	4.70		
	C	→3)-β-D-Gal*p*-(1→	C	109.54	72.23	82.20	69.67	68.37	62.74	
			H	4.36	4.18	3.74	3.67	3.68	3.96	
